# From Ethnic Herbal Medicine to Unani Traditions: Medicinal Plant Knowledge across Southwestern China and Northern Pakistan

**DOI:** 10.12669/pjms.42.9.20097

**Published:** 2026-09

**Authors:** Gao Jian, Xu Ziheng, Qu Qiumei

**Affiliations:** 1 Gao Jian Associate Professor, College of The Humanitiesm, Harbin Normal University, Harbin, China; 2 Xu Ziheng Associate Professor, School of Economics and Management, Harbin Normal University, Harbin, China; 3 Qu Qiumei Visiting Lecturer, Department of History & Pakistan Studies, University of the Punjab, Lahore, Pakistan

**Keywords:** Southwest China, Northern Pakistan, Ethnic Herbal Medicine, The Unani Medical Tradition, Exchange of Medical Knowledge

## Abstract

Ethnic herbal medicine originated from the medical knowledge accumulated by ethnic groups in their struggle against disease and harsh environments; the history of each ethnic group’s development through long-term coexistence and competition with nature is, in itself, a history of medicine. In northern Pakistan, herbal plants thrive and are readily available, and medicinal plants exhibit an exceptionally rich biodiversity. Yunnan’s unique natural environment and biodiversity have fostered a wealth of ethnic medicine and cultural resources, making it a treasure trove of global medicine and culture. There is significant potential for cooperation between Yunnan’s border ethnic regions and northern Pakistan in areas such as pharmaceutical research and development, joint construction of pharmaceutical plants, training of medical professionals, and the exchange of medicinal plant cultivation techniques. This topic was chosen to explore the similarities and cultural significance of traditional medicinal plant knowledge between the ethnic herbal medicine systems of Southwestern China and the Unani medical traditions of Northern Pakistan.

***This study is part of the research project entitled “General Project of Philosophy and Social Sciences in Heilongjiang Province “A Study on the Pragmatic Motivation and Mechanism of ‘Sub-Prototypical Category Fuzzy Expressions’ in Modern Chinese” (22YYB250)” and” “Heilongjiang Provincial Language Commission Project (General Project): Research on Chinese Character Teaching and Cultural Communication from the Perspective of International Chinese Education (2026Y064)”***

## INTRODUCTION

A total of 6,559 species of natural plant and animal medicinal resources have been identified in Yunnan, with 4,000 to 5,000 species suitable for medicinal use,^1^ Only 600 to 700 plant species in Pakistan are used for medicinal purposes.^2^

Yunnan and Pakistan have extensively engaged in exchanges and cooperation across various fields, including traditional medicine technology, medical education, and the training of medical professionals. This not only fosters mutual understanding and trust but also helps Pakistan cultivate talent and enhance its capacity for scientific and technological innovation, thereby strengthening its own development capabilities. Furthermore, it facilitates joint efforts with Pakistan to advance the development of the traditional medicine industry, explore and develop medical resources, and preserve and pass down traditional medical texts, while jointly promoting the construction and development of ethnic regions and maintaining regional harmony and stability. One limitation of this study is that the documentation of ethnobotanical data vary across regions, which may affect the comprehensiveness and comparability of the findings.

## METHODOLOGY

This paper employs methods such as literature review, ethnobotanical investigation, and comparative analysis. Although there is an abundance of quantitative study on this issue, there are still few qualitative studies on related topics. As a result, the main source of information for this study is a review of the literature, which includes regional surveys of medicinal plants, ancient medical writings, and ethnobotanical investigations. Ethnobotanical techniques, cross-cultural analytical frameworks, disease classification assessments, and comparisons of medicinal plant applications are all examined using a comparative analytical approach.

### Comparison of Medicinal Plants and Their Therapeutic Uses

### Different plants for the same condition:

In Pakistan, willow bark is used to treat osteoarthritis, muscle pain, gout, and headaches or pain caused by menstrual cramps.^3^ Aristolochia frutescens, a traditional ethnic medicine from Southwest China, it is used to treat edema, beriberi, and rheumatoid arthritis.^4^ Yanda is used to clear heat and cool the blood, regulate menstruation and relieve pain, and treat contusions and bruises.^4^ Bamboo-leaf Orchid can be used to treat hepatitis, traumatic injuries, rheumatic pain, and venomous snake bites.^5^ Ground Sandalwood is used to treat rheumatism and frostbite.^6^ The fruit and roots of the snotberry are used to treat fractures and bruises.^7^ The lacquer tree can help treat rheumatic diseases.^5^

**Table:** Comparative Ethnobotanical Knowledge Systems: Southwestern China vs Northern Pakistan.

Core Theory	Topic Categories	Ethnic Medicine of Southwest China	Unani Medicine in Northern Pakistan	Explanatory Memorandum
1. Knowledge Generation Model	Experience-driven vs. Theory-driven	Observing the efficacy of plants, trial-and-error experiences, and foraging knowledge	Classical medical theory (the Hippocratic-Galenic system), humoral theory	China tends toward “empirical induction,” while Pakistan tends toward “theoretical deduction.”
		Symptoms—Plant Correspondences	Constitution—Logic of Balance and Regulation	There are fundamental differences in the logic of knowledge generation
2. Knowledge Framework	Classification Logic	Classification by thermal characteristics, local ecological classification	The Four Humors Theory (blood, phlegm, yellow bile, black bile)	Classification systems reflect cultural cognitive structures
		Classification by function (pain relief, detoxification)	Systematic Medical Classification (Organ + Constitution)	There are significant differences in the degree of structuring
3. Methods of Knowledge Transmission	Oral Tradition vs. Written Tradition	Oral tradition, family transmission, and the knowledge of traditional healers	Medical texts (Unani texts), apprenticeship	The degree of documentation determines the stability of knowledge
		Ritual memory	Canonization	Pakistan’s system is more stable
4. Healthcare System	Combination of Treatment Modalities	Herbal decoctions, topical applications, and ritual healing	Herbal Medicine + Dietary Therapy + Bloodletting	Both are “multimodal treatment systems”
		A blend of witchcraft and religious healing	Spiritual Healing	Varying degrees of religious integration
5. Integration of Religious Culture	Impact of Belief Systems	Influences of Tibetan Buddhism, Nature Worship	The Ethical Framework of Islamic Medicine	Religion Shapes the Legitimacy of Medicine
		Belief in Mountain Gods/Nature Spirits	spiritual healing	The boundaries of medicine and religion differ
6. The degree of medical institutionalization	National/Institutional Integration	Policies on Ethnic Medicine Incorporated into the Traditional Chinese Medicine System	The Unani medical system is recognized by the state	Both have been institutionalized, but through different pathways
		Intangible Cultural Heritage	Medical School/Pharmacopoeia System	China focuses on cultural preservation, while Pakistan emphasizes the systematization of medicine
7. Knowledge Dissemination Channels	Mechanisms of spatial diffusion	Mountain Community Networks, Local Exchange	The Southern Route of the Silk Road and the Islamic Medical Network	Different spatial transmission patterns
		Spread among villages	Academic-Religious Communication Networks	Pakistan is more transregional
8. The Logic of Ecological Adaptation	The Environment-Knowledge Relation-ship	Dependence on Medicinal Plants in Alpine Forests	Mountain and plain ecosystems	The environment determines drug diversity
		Seasonal Harvesting Tips	Herbal Medicine Trading Supplement System	Different methods of obtaining resources
9. The Modern Transformation of Knowledge	The Impact of Modernization	Knowledge Loss + Return to Tradition	Standardization + Commercial Expansion	Both sides have undergone modernization
		Strengthening of ethnic identity	Marketization of Healthcare	Different directions for transformation
10. Cultural Perception Models	Differences in worldview	Animism	The Cosmology of Fluid Balance	Medicine Reflects a Cosmological View
		The Unity of Humanity and Nature	A Medical Perspective on the Body’s Systems	The Core of Differences in Cognitive Structures

Descriptions of digitalis in Unani literature, its leaves possess pharmacological effects such as cardiotonic, diuretic, sedative, hemostatic, and antipyretic properties, and are used to treat a variety of conditions, including left ventricular hypertrophy, palpitations, ascites, nosebleeds, hemoptysis, menorrhagia, epilepsy, pneumonia, chronic pleurisy, as well as trauma and burns.^6^ Hairy-flowered Plantain is to treat pneumonia and appendicitis; for external use on skin infections, crush the plant and apply the paste to the affected area.^4^ Eupatorium is used to treat meningitis, Japanese encephalitis, pneumonia, erysipelas, sore throat, and skin rashes.^4^ Western Green Pod Leaf is use to treat dysentery. For external use on burns, grind the herb into a powder, mix with egg white, and apply to the affected area.^4^ The bulbs of the giant lily are used to treat hemoptysis caused by tuberculosis and high fever in children; the fruit is used as a medicinal ingredient in Yunnan’s Damaodouling formula.^5^ Classical Unani medical texts describe white poppy seeds have been used to treat a variety of ailments, such as diarrhea, dysentery, coughs, premature ejaculation, chronic coughs, insomnia, and skin conditions, and are also consumed as a nutritional food.^7^

### The Same Plant Treats Different Diseases:

Ancient Chinese scholars were among the first to provide a comprehensive record of the medicinal uses of willow bark. The *Shennong Bencao Jing* states that the roots, bark, branches, and leaves of the willow tree can all be used medicinally; they have the effects of expelling phlegm, improving vision, clearing heat, detoxifying, promoting urination, and warding off wind. When applied topically, they can treat toothaches. The *Compendium of Materia Medica* states that willow leaves, when decocted, can treat internal bleeding in the chest and abdomen, relieve pain, and cure scabies; willow branches and root bark, when boiled in wine, can be used as a mouthwash for toothaches, and when decocted and taken orally, can treat jaundice and leukorrhea; willow catkins can stop bleeding, treat spleen dampness, and relieve spasms in the limbs. The *Compendium of Chinese Herbal Medicines* describes foxglove as a cardiac stimulant. Its primary effects include stimulating the heart muscle, increasing myocardial contractility, significantly enhancing stroke volume, and improving blood circulation. It also has a diuretic effect in patients with cardiac edema.

### A Cross-Cultural Perspective on Traditional Medicine

### The Influence of Traditional Ethnic Medicine:

Ethnic medicine is emerging as a vital source and focal point for innovative drug research worldwide.^8^

Ethnic medicine is an important public welfare undertaking and the most distinctive pillar industry in ethnic regions. For a long time, the Party and the government have attached great importance to the development of healthcare and the ethnic medicine industry in ethnic regions, formulating and implementing a series of policies and regulations that have enabled significant progress in these sectors. The Yunnan Provincial Government has successively issued a series of policies and regulations, including the “Yunnan Provincial Regulations on Traditional Chinese Medicine,” the “Implementation Opinions on the Implementation of the Strategic Plan for the Development of Traditional Chinese Medicine (2016–2030) in Yunnan Province,” and the “13th Five-Year Plan for the Development of Traditional Chinese Medicine.”

### The Influence of Unani Medicine and Islamic Medicine:

In Unani medicine, medicines and foods are also classified according to their temperaments, as they have different chemical compositions. The temperament of the human body is determined by the “humors” (that is, the four bodily fluids), which are themselves composed of different elements.^9^

From a cross-cultural perspective, although traditional ethnic medicine in Southwest China and Unani traditional medicine in northern Pakistan originated from different civilizational systems, they share distinct “common logics” in terms of knowledge structures, conceptions of nature, and health philosophies, while also exhibiting their own unique developmental trajectories.

These common features are manifested in the following ways: both reject the “single-cause theory” of disease and emphasize systemic balance; both stress the vital role of the natural state in health; both belong to “empirical medical traditions” at varying stages of development; and both represent low-cost yet viable forms of healthcare. In addition, traditional medicine of the ethnic groups in Southwest China and Unani traditional medicine in northern Pakistan share distinct similarities in the “integration of spirituality and medicine.”

### Of course, structural differences also exist:

- Differences in the maturity of theoretical systems, variations in knowledge dissemination methods, and distinct cultural backgrounds (ethnic medicine: a context of multi-ethnic and ecological cultures; Unani medicine: the Greco-Arabic-Islamic civilizational system).
- In summary, ethnic medicine leans toward ecology and local experience, while Unani medicine leans toward philosophy and systemic theory.

### Cultural Ties Between Chinese and Pakistani Traditional Medicine-

Traditional medicine plays a unique role as a cultural bridge in the construction of the China-Pakistan Economic Corridor.

First, they can effectively promote cultural exchange and mutual understanding, enhance cultural identity within the “China-Pakistan Economic Corridor,” and create a more harmonious and diverse cultural environment.

Second, they contribute to the development and innovation of traditional medicine itself. The opening up and cooperative exchange of traditional medicine can revitalize the field, creating an interactive platform where traditional medicine from China and Pakistan can learn from, absorb, complement, and refine one another, thereby bringing more opportunities for development and innovation. At the same time, it can effectively promote the intrinsic connection and integration of traditional medicine in both countries, driving the industrialization and internationalization of traditional medicine, and further enhancing its strength and influence.

Third, The peoples of China and Pakistan enjoy a very friendly relationship. On issues such as developing new indigenous medicines, introducing and cultivating valuable medicinal and aromatic plants, establishing quality standards for herbal materials and plant-based preparations as soon as possible, strengthening research on herbal preparations, and better integrating traditional and modern medicine to promote the development of national healthcare and the pharmaceutical industry, Pakistani counterparts look forward to receiving assistance from their Chinese counterparts and engaging in broader and deeper cooperation.

## CONCLUSION

This study found that although traditional medicine in Southwest China and Unani traditional medicine in northern Pakistan originated from different civilizational systems and share common characteristics as well as structural differences in certain aspects, there remains significant room for collaboration between the two.

How Pakistan can better integrate its traditional medicine with modern medicine, and engage in broader and deeper cooperation with China, has become a key focus for future medical collaboration between China and Pakistan.
